# 25+ Years of Research on Nonphotochemical Laser-Induced Nucleation (NPLIN)

**DOI:** 10.1021/acs.cgd.5c00266

**Published:** 2025-04-24

**Authors:** Bruce A. Garetz, Ryan L. Hartman

**Affiliations:** Department of Chemical and Biomolecular Engineering, Tandon School of Engineering, New York University, Brooklyn, New York 11201, United States

## Abstract

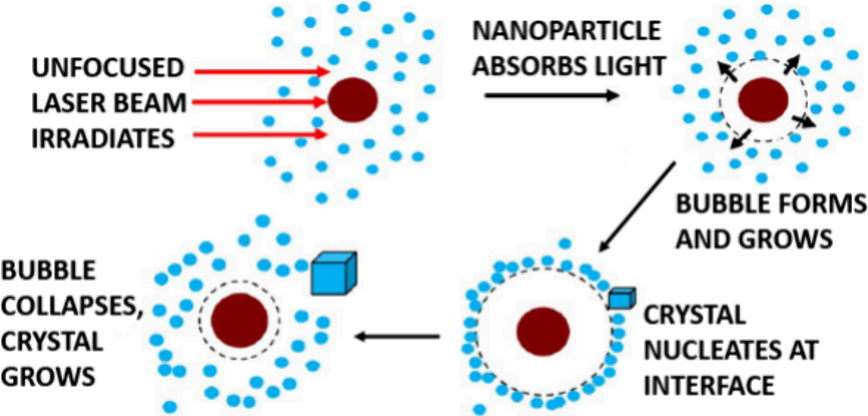

This Perspective looks back on 25+ years of research on nonphotochemical laser-induced nucleation (NPLIN), focusing in particular on the accidental discovery of the phenomenon, a discussion of the three proposed mechanisms for NPLIN, the application of microfluidics to the study of NPLIN, industrial application of NPLIN, and the future of NPLIN. It also discusses the recent finding that high pressure suppresses NPLIN, and how this finding provides the strongest evidence to date for the impurity heating mechanism for NPLIN.

## Abstract 2

Looking back at 25+ years of research on nonphotochemical laser-induced nucleation, including its accidental discovery, the proposed mechanisms, the application of microfluidics, its industrial applications, and its future.

## Introduction

Crystallization is an important process in industry for isolating and purifying chemical substances, such as pharmaceutical drugs, dyes, fertilizers, explosives and other specialty chemicals.^1^ Primary nucleation is the formation from solution of a thermodynamically stable solute cluster (the critical nucleus) that has the same ordered structure as the nucleated crystal, and which eventually grows into a macroscopic crystal.^2^ Many solutes can organize into different crystal structures known as polymorphs. Different polymorphs have different physical and chemical properties, such as solubility, morphology, melting point, dissolution rate, density and bioavailability, so the control of polymorphs is important in the pharmaceutical industry.^3^ Nearly 30 years ago, one of us (Garetz) accidently discovered that exposing a supersaturated solution to unfocused near-infrared laser pulses could reduce the nucleation time from days or weeks to seconds.^4^ Recently, laser-induced nucleation has been singled out as an important tool in the arsenal of industrial crystallization.^5^

The letter one of us (Garetz) published in the first issue of *Crystal Growth& Design* was our second publication on the subject of nonphotochemical laser-induced nucleation (NPLIN).^6^ The first paper on NPLIN was published 5 years earlier, in *Physical Review Letters* in 1996.^4^ In that paper, we reported on the accidental discovery that irradiating supersaturated aqueous solutions of urea with unfocused millijoule nanosecond near-infrared laser pulses could induce the nucleation of crystalline urea within seconds, compared to days or weeks without the laser. There was a preexisting literature concerning the ultraviolet-light induced nucleation of supersaturated vapors dating back to the 19th century,^7^ and the evidence suggested that this phenomenon was caused by the photochemical reaction of gaseous trace impurities in the sample leading to clusters that acted as the sites for heterogeneous nucleation.^8^ Because the urea solution was essentially transparent at the laser wavelength, and because the near-IR photon energy was too small to induce photochemistry, we called the phenomenon nonphotochemical laser-induced nucleation (NPLIN) to distinguish it from the earlier UV/gas-phase studies.

The letter published in the first issue of *Crystal Growth& Design* reported on the NPLIN of supersaturated aqueous solutions of glycine. Unlike urea, glycine had the additional complexity that it could crystallize into three different polymorphs, known as α-, β-, and γ-glycine.^9^ When glycine nucleates spontaneously from aqueous solution, the α-polymorph is formed. The most stable form is the γ-polymorph which crystallizes spontaneously from aqueous solution under either acidic or basic conditions. Surprisingly, the NPLIN of aqueous glycine produced the γ-polymorph rather than the α-polymorph, which meant that NPLIN could provide some control over polymorph formation.

Since those two papers were published, there have been nearly 50 papers published on NPLIN by numerous research groups, with the majority of them appearing in *Crystal Growth& Design*.^10^ NPLIN has been observed in supersaturated solutions of small organic molecules, inorganic salts, gases, and proteins, and in supercooled melts.^11−15,17^ The first several NPLIN papers also spawned a range of laser-induced nucleation studies using different laser types and conditions: using *focused* nanosecond, picosecond and femtosecond pulses, producing much more extreme laser intensities (GW/cm^2^) giving rise to nucleation resulting from optical breakdown, cavitation and shock waves;^18−21^ using 1-W near-IR continuous-wave (cw) laser beams or high-repetition-rate femtosecond laser beams, tightly focused at the air/solution interface of a thin-film droplet consisting of undersaturated, saturated or supersaturated solutions, trapping solute clusters through optical gradient forces (same as those used in laser tweezers), eventually inducing nucleation of the solute.^22−26^

Some of the important features of NPLIN are (1) it occurs in a wide variety of substances,^11−17^ (2) it is not strongly dependent on laser wavelength,^27^ (3) it provides control over the time and place that nucleation occurs,^28^ (4) it provides some control over polymorphism,^6,11,12,29,30^ (5) it exhibits an intensity threshold below which no nucleation occurs,^4^ (6) the probability of nucleation increases with increasing supersaturation and increasing laser intensity,^30^ (7) it exhibits some dependence on laser polarization,^4,11,12,27,29,32^ although there is some disagreement about this,^30,33,34^ (8) aging of the solution is required in some systems, e.g. glycine,^35^ but not in most others, and (9) filtration of the solution reduces the probability of nucleation,^36,37^ while addition of nanoparticles increases the probability of nucleation.^38^ NPLIN can be performed in closed systems so there is less chance of contamination. Typical laser peak intensities used are 10–200 MW/cm^2^, and typical threshold intensities are 5–50 MW/cm^2^.

This paper is not intended to be an exhaustive review of the research that has been published on NPLIN, as there are several good reviews already published.^10,39,40^ It is meant to be a personal perspective on NPLIN research, one of us (Garetz) having been involved in it from the very beginning and both of us continuing to study it today, and it will focus on (1) how we accidently discovered NPLIN, (2) the proposed mechanisms of NPLIN, (3) how microfluidics is revolutionizing NPLIN, (4) the pressure dependence of NPLIN, (5) process design and industrial application of NPLIN, and (6) the future of NPLIN.

## An Accidental Discovery and the Optical Kerr Mechanism of NPLIN

The conversation that ultimately led to the accidental discovery of NPLIN was one I (Garetz) had with my colleague at the time at Polytechnic University (now the NYU Tandon School of Engineering), Allan S. Myerson. I was interested in laser spectroscopy and nonlinear optics, and Allan was interested in crystallization. Sometime in 1993, Allan called me into his office and pointed out to me that nobody had ever observed a subcritical molecular cluster or a critical nucleus during the primary nucleation process. He wondered if I could think of a way to use lasers or optics to observe one. I came up with a proposal to look for second harmonic generation (SHG) from urea subcritical molecular clusters in a supersaturated aqueous urea solution. Classical nucleation theory indicates that these clusters will be in the same arrangement as a nucleated crystal.^2^ SHG is a nonlinear optical phenomenon in which the interaction of an intense incident laser beam at frequency, ω, with a noncentrosymmetric medium induces the generation of light at twice the incident frequency, 2ω.^41^ A supersaturated urea solution is centrosymmetric, so would not produce SHG, whereas a subcritical urea cluster is noncentrosymmetric (based on classical nucleation theory), so could generate scattered light at twice the incident frequency. I had a Quanta-Ray Q-switched Nd:YAG laser in my lab that produced nanosecond pulses of near-infrared light at a wavelength of 1064 nm, so that by irradiating a supersaturated urea solution with pulses from this laser, we could look for scattered green SHG light at half the laser wavelength, 532 nm.

The proposed experiment was fairly simple: prepare a sealed glass vial filled with a supersaturated aqueous urea solution, expose it to pulses from the Nd:YAG laser, and look for green scattered light. I assigned this task to an undergraduate chemistry major, Rod Young,^42^ who was aided by my doctoral student, Janice Aber. It took them a while to figure out a repeatable procedure for preparing supersaturated solutions that could survive for days without spontaneously nucleating. Rod was then to expose these solutions to the laser and to try to detect green scattered light using filters and photodetectors. After struggling with this detection system without success, Rod used the human eye as a fallback detection system. Wearing safety goggles that blocked 1064 nm light but transmitted green light, Rod was to look for green scattered light by eye, something I have at times described as a 17th-century style experiment. This experiment was revisited by Ward et al. in 2013.^43^

At some point, Rod came to my office disappointed, saying that he had not seen any green light, and that every time he tried increasing the laser intensity by turning on the laser amplifier, needle-shaped crystals would form, preventing him from continuing the experiment. I was not at all disappointed, thinking that perhaps we had stumbled onto something interesting. The main problem was that the urea solution was essentially transparent at the incident laser wavelength, so how was the light interacting with the solution? While water does absorb light weakly in the near-IR, the effect of this absorption would be to raise the temperature of the solution a few degrees. Because the solubility of urea increases with increasing temperature, heating would make it more stable and less likely to nucleate. It occurred to me that there were a variety of nonlinear optical phenomena that did not involve the absorption of photons (e.g., SHG), but depended on the intense oscillating electric field associated with the light, and the electric field strength of the laser pulses used in our experiments was quite high, on the order of 10^7^ V/m. One such phenomenon is the optical Kerr effect,^41^ in which an intense laser pulse incident on a liquid such as carbon disulfide could induce the CS_2_ molecules to tend to align in a direction parallel to the electric field vector of the light, inducing the liquid to become birefringent. This effect requires the molecules to have an anisotropic shape and polarizability, and the most polarizable axis would tend to align in the direction of the field. We proposed that in our supersaturated solutions, the electric field of the light was aligning urea molecules in prenucleating clusters, helping those clusters to organize into an ordered crystalline structure by lowering the entropic part of the activation free energy to nucleation. Unfortunately, the calculated energy of interaction between the optical electric field and a urea molecule was orders of magnitude too small to sufficiently reduce the activation barrier to nucleation to match the experimental observations. We proposed that cooperative effects among solute molecules could enhance the interaction energy,^32^ but simulations by Knott et al. suggested it was still not nearly enough.^44^

## Dielectric Polarization Mechanism of NPLIN

We pretty much had the NPLIN field to ourselves until around 2006, when Andrew Alexander at the University of Edinburgh began to study it (see his recollections in his *Journal of Chemical Physics* Perspective, ref (39), p 4). All of our early studies involved organic molecular solutes, and Alexander was the first to study inorganic salts. In 2009, Alexander and Camp reported the observation of NPLIN in aqueous KCl.^45^ Because KCl consists of spherical ions and crystallizes into a cubic (and hence isotropic) crystal structure, there are no anisotropic particles to align in solution, and the Optical Kerr (OK) mechanism would predict no enhancement of nucleation in an optical electric field. They proposed an alternative electric-field—induced-dipole model they called the Dielectric Polarization (DP) model. It was based on the fact that in aqueous KCl, a subcritical cluster would have a larger relative permittivity than the surrounding solution. Their model accounts for the electronic polarization of the cluster (which all materials exhibit) rather than the nuclear orientational polarization (which only anisotropic molecules exhibit) that was treated in the OK model. An oscillating electric field from the laser would induce an oscillating polarization in the cluster. The interaction of this polarization with the field results in a lowering of the free energy of the cluster. The solution would undergo a similar lowering of its free energy, but if its relative permittivity is lower than that of the cluster, this would result in a stabilization of the cluster relative to the solution by an amount proportional to Δ*ϵE*^2^, where Δϵ is the difference between the relative permittivity of the cluster and that of the solution, and *E* is the electric field amplitude, reducing the barrier to nucleation. At high enough laser intensity, the cluster would become critical and would nucleate. This model also predicts that the number of crystals nucleated should be proportional to the laser intensity, which has been confirmed in a variety of studies.^13,31,35^ On the other hand, it does not predict the threshold intensity, below which no nucleation occurs, that has been experimentally observed in most systems. It also does not explain the nucleation of CO_2_ bubbles from the NPLIN of carbonated water, where the relative permittivity of a gaseous CO_2_ cluster is lower than that of the solution.^46,47^

## Impurity Heating Mechanism

**Figure 1:**
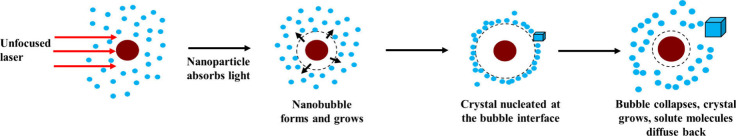
Impurity heating model. Sequence of events during the laser-heating of a nanoparticle. Red circle represents a nanoparticle, blue dots represent solute molecules or clusters, dotted circle represents the bubble/solution interface.^38^

Starting around 2015, there were a series of reports that filtration of solutions before laser irradiation reduced the probability of nucleation.^36,37^ Further reports showed that addition of nanoparticles to supersaturated solutions could enhance the NPLIN probability.^37,38^ Analysis of some of the filtrates indicated the presence of iron oxide particles, which would strongly absorb near-IR and visible light. This implied that there were mesoscale particles in the solution that were important for NPLIN, possibly solute clusters or impurity particles. This gave rise to the impurity heating (IH) mechanism, in which an impurity particle would absorb laser light, heating it by hundreds of degrees. This heat could be transferred to the surrounding solution, inducing the formation of a solvent vapor bubble, a process known as thermocavitation. As this bubble expands, any solute molecules in the path of the expanding interface would be pushed outward and concentrated at the bubble/solution interface. The local supersaturation at the interface would be higher than that of the bulk solution, inducing a higher rate of nucleation (see Figure 1). This was put into an explicit model form by Ward et al.,^47^ which predicts the size of the bubble produced from a nanoparticle of a particular size and absorption efficiency when exposed to a laser pulse of a given intensity and duration in a given solvent. In particular, one needs to know the spherical particle radius and the complex refractive index of the material, from which one can calculate using Mie theory how much light energy the particle absorbs from a laser pulse of a given wavelength, intensity and duration.^48^ The 15 nm-radius iron (II–III) oxide impurity particles used in our recent NPLIN experiments^38^ will not focus visible or near-infrared light because their size is much smaller than the wavelength of the incident light. Such a particle will scatter the light, but will not focus it. Furthermore, because such a particle is highly absorbing at those wavelengths, essentially all of the incident light will be absorbed, and very little will be scattered.^48^

We can estimate the increase in concentration of the solute caused by a vapor bubble using a simple geometrical argument. Assuming for the moment that the radius, *r*, of the induced bubble is much larger than that of the absorbing nanoparticle, and assuming that the solute that is swept up at the bubble interface undergoes diffusion such that it is confined to a spherical shell surrounding the bubble (mass-transfer boundary layer) with thickness δ, then the solute concentration will be increased by a factor, *f*, of the ratio of the bubble-plus-shell volume to the shell volume alone.
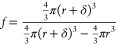
1

In our experiments at ambient pressure, based on the absorption efficiency of a 15 nm radius iron (II, III) oxide nanoparticle, a peak laser intensity of 20 MW/cm^2^, a laser pulse duration of 6 ns, and on the thermodynamic properties of water, the estimated bubble radius is 168 nm.^38^ The shell thickness depends on the extent to which the solute molecules have diffused away from the interface and is a function of time. It can be approximated by (*Dt*)^1/2^, where *D* is the solute diffusion coefficient and *t* is time.^38,49^ For KCl (aq) after 1 μs, δ = 44 nm, and using eq 1, the local concentration is increased by a factor of 2.0. However, this does not mean that the supersaturation is increased by a factor of 2, because the temperature is simultaneously changing, and the supersaturation depends on the temperature. During the thermocavitation process, the temperature rises at early times and falls back to room temperature at later times. As the temperature falls, the vapor bubble collapses (see Figure 1), inducing pressure waves that can also concentrate solute molecules and may also be responsible for nucleation.^50^

The impurity heating model potentially explains the threshold behavior of NPLIN, as one can imagine that below a certain laser intensity, the amount of heating would be insufficient to generate a bubble, or that the generated bubble would be too small to induce nucleation. It also explains the weak wavelength dependence of NPLIN, since many impurity particles can have broad absorption bands.

## Microfluidic NPLIN

**Figure 2:**
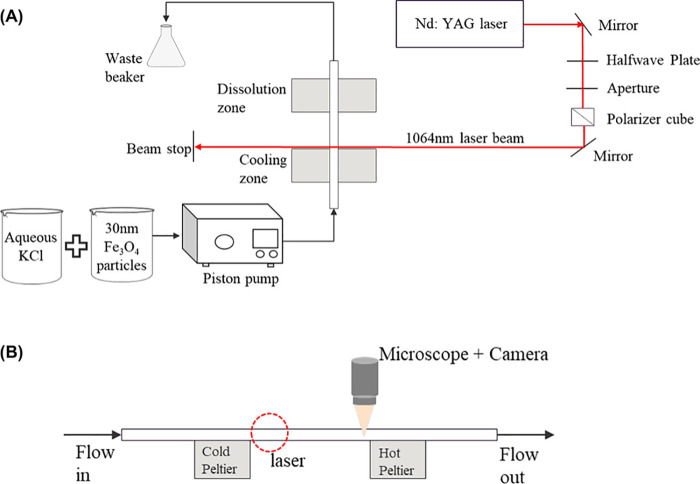
Microfluidic NPLIN setup. (A) shows the view from above. (B) shows the view from the side.^38^

**Figure 3:**
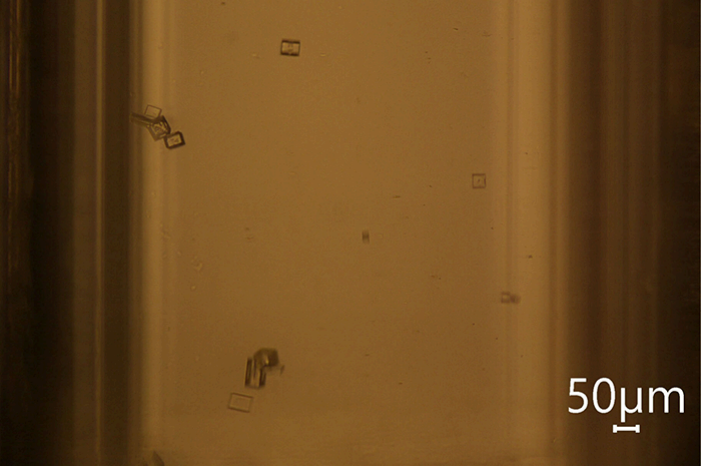
Frame from a video of KCl crystals flowing through 1 mm square capillary at 300 μL/min.^38^

**Figure 4:**
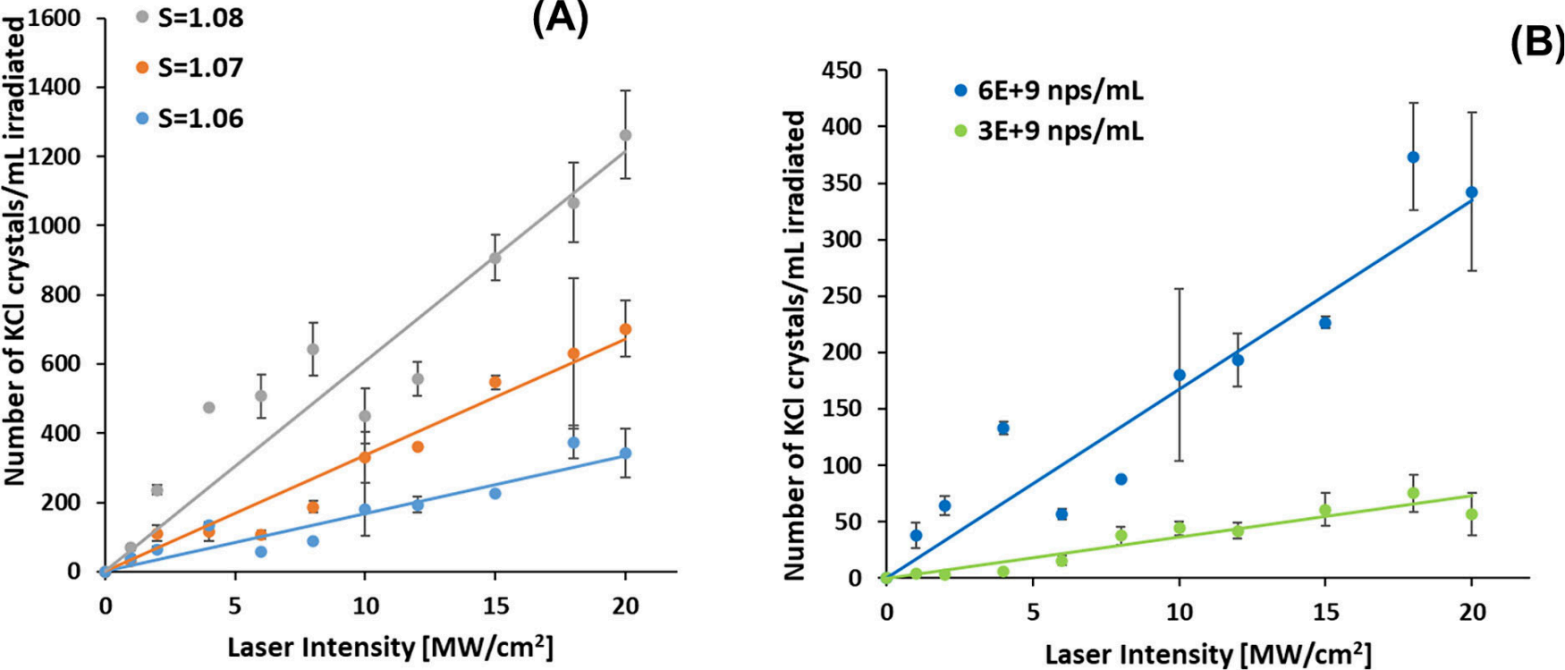
Plots of *N*_crystal_ vs laser intensity for the NPLIN of aqueous KCl doped with Fe_3_O_4_ nanoparticles. (A) shows the dependence on supersaturation, *S*. (B) shows the dependence on nanoparticle concentration.^38^

The most important technical advancement in NPLIN studies, in our opinion, has been the application of microfluidics to NPLIN.^31,35,38,51^ In around 2017 we received some seed funds from the NYU Materials Research Science and Engineering Center (MRSEC) to look into carrying out NPLIN experiments in microfluidic devices. This proved to be a turning point in the quantification of NPLIN experiments. Up until then, an NPLIN experiment involved filling a large batch of milliliter-sized glass vials with identical supersaturated solutions, exposing each of them to laser pulses, counting the number of vials that generated crystals, and calculating the probability of nucleation. Then one studied how that probability changed with laser intensity or supersaturation. There were some efforts to automate this process using carrousels containing multiple vials,^40^ but it was still the same basic batch process. With continuous flow microfluidics (see schematic of layout in Figure 2), one does not need to calculate the probability of forming a crystal; one can actually count the crystals that form during continuous flow. If the nucleation rate is low, then one just needs to increase the run time of the experiment so that a greater volume of solution is exposed to the laser. Since the laser beam cross section is larger than the microfluidic channel width, every volume element of solution that flows through the channel is exposed to laser light, in contrast to milliliter-sized vials, where typically only a few percent of the solution volume is exposed to the laser. Because of the small solution volumes used in microfluidics, and because of excellent and well-defined mass and heat transfer, it is possible to pump an undersaturated solution into the channel and to create supersaturation on-chip by cooling the flowing solution with a Peltier cooler in good thermal contact with the microfluidic device. In other words, we are able to impose a step input in the local fluid temperature. While earlier work by us and others had to plot probability of nucleation vs the parameter being studied (laser intensity, supersaturation, etc.), with microfluidics, one can plot the actual number of crystals observed per unit volume of solution that has been irradiated vs the parameter of interest. They can be counted by mounting a microscope and camera downstream from the irradiation zone and making a video recording of the flowing crystals (see Figure 3). Just to provide an example, we recently reported the effect on NPLIN of doping aqueous KCl with Fe_3_O_4_ nanoparticles. In order to apply the IH model of Ward et al., we took this model one step further by assuming that the probability of nucleation caused by a nanobubble would be proportional to the bubble volume. With this additional assumption, we predicted that the number of crystals formed should be proportional to both the laser intensity and the nanoparticle concentration. Figure 4 shows plots of the number of crystals observed per mL of solution exposed to the laser versus the laser intensity. Those plots are linear, which is consistent with both the DP and the extended IH models. In part A, we see that with increasing supersaturation of KCl, the slopes of the straight lines increase. In part B, we see that doubling the concentration of nanoparticles roughly doubles the slope of the straight line.

There are pragmatic considerations with microfluidic NPLIN. If the channels are too small or the supersaturation is too low, the rate of nucleation could become too low to observe any crystals in a reasonable period of time. If the flow rate is too slow and the crystals are growing too quickly, it is possible to get blockage of the channels.^52^ The designed residence time should match the growth kinetics. Not all systems are amenable to on-chip cooling to achieve supersaturation. An example of this is aqueous glycine, where aging appears to be necessary after supersaturation has been achieved, possibly to allow a population of mesoscale glycine clusters to develop, which is consistent with a two-step nucleation process.^36,53^ In the case of glycine, we created the supersaturation off-chip and allowed the solution to age before pumping it into the microfluidic channel.^35^ For crystallizations that are solvent induced, one may design the injection of cosolvent(s) axially along the flow in the main microchannel, which has been done for seeding in other microfluidic crystallizations.^54^

Korede et al. also carried out NPLIN on Fe_3_O_4_-doped aqueous KCl, but using a two-phase droplet-based microfluidic setup, as opposed to the continuous-flow setup used in our studies, in which supersaturated aqueous KCl droplets were dispersed in a continuous phase consisting of silicone oil.^51^ This approach has several disadvantages compared to the single-phase flow approach. Using slugs is the microfluidic equivalent of a batch system. Each slug is a separate crystallizer. Since the slug volume was very small, on the order of 1.2 μL, the rate of nucleation, which is proportional to the slug volume,^55^ is also very small, such that one is likely to observe zero or one crystal per slug, similar to the situation found when using vials. Therefore, one is restricted to calculating the probability of nucleation rather than counting the number of crystals formed. In addition, the slugs have a large surface to volume ratio, so that the probability of heterogeneous nucleation at the droplet/oil interface is substantial, essentially adding noise to the measurement of the probability of nucleation. This noise was so great in ref (48). that it completely masked the presence of the threshold below which NPLIN does not occur. In single-phase microfluidics, this kind of noise was completely absent, as we observed no spontaneous or heterogeneous nucleation when flowing solutions through the microfluidic channel with the laser turned off.^38^

Design of *in situ* measurements indeed is a tremendous advantage of the experimental approach that also enables computer-aided high-throughput screening. Micrographs and other data obtained in real-time microfluidic NPLIN can be analyzed by machine learning algorithms to correlate the crystallization conditions to the observed crystal characteristics. But, perhaps one of the most important applications of microfluidics is their safe operation at elevated pressures. Together, *in situ* measurements and high-pressure microfluidics create the possibility of rapid exploration of NPLIN mechanisms and quantitative model development.

## Pressure Dependence of NPLIN

**Figure 5:**
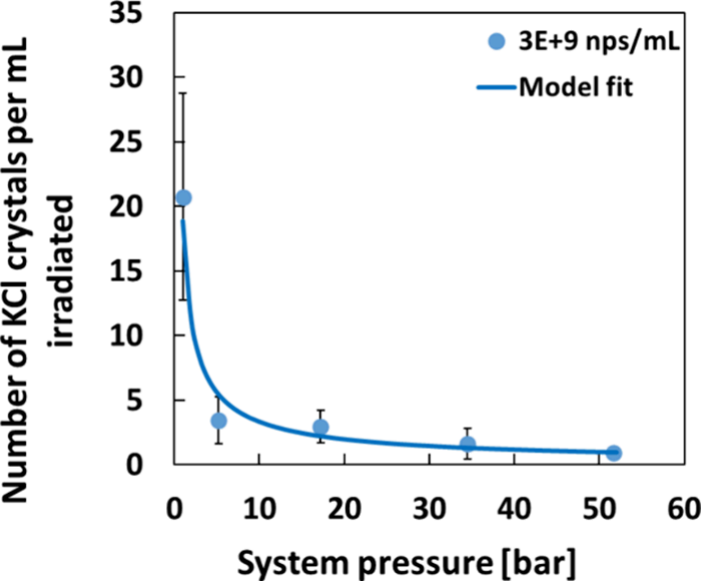
Plot of *N*_crystal_ vs pressure for the NPLIN of aqueous KCl doped with Fe_3_O_4_ nanoparticles. The KCl supersaturation ratio was 1.06, and the peak laser intensity was 20 MW/cm^2^. Solid line is the fit to the IH model (Equation 2).

In 2018, I (Garetz) gave a talk on the latest developments in NPLIN at an X-ray Workshop held in the NYU Chemistry Department, where I mentioned the three competing models for NPLIN. One of the other speakers was J. Michael McBride, a physical organic chemist from Yale. Over lunch after my talk, in light of the proposed IH model involving bubble formation, Mike asked if anyone had studied the pressure dependence of NPLIN. I said that it hadn’t been studied, but thought that the suggestion was an intriguing one. If solvent vapor bubble formation is necessary for NPLIN, then if one raises the pressure above the critical pressure of the solvent, bubble formation becomes thermodynamically forbidden, and no crystals should form. At the time, the idea of looking into this was not really feasible in vials. Several years later, after we had had some success with microfluidic NPLIN, the idea reemerged, and one of us (Hartman) had experience in operating microfluidic devices at high pressures. It requires pumps, tubing, channels and connectors that can withstand the high pressure, and the addition of an in-line pressure regulator downstream from the microfluidic device. We recently reported on very strong evidence that the primary mechanism of NPLIN is the IH model, in which laser light absorbed by nanoscale impurity particles heats up the surrounding solvent sufficiently to form solvent vapor bubbles, and nucleation occurs at the bubble/solution interface. This was done by carrying out NPLIN in a microfluidic device as a function of pressure, and showing that NPLIN is suppressed at pressures above ambient pressure.^56^ At 51.7 bar, the crystal yield of NPLIN was reduced to approximately 5% of the yield at ambient pressure, and the plot of *N*_crystal_, the number of crystals observed per mL of irradiated solution, vs pressure (see Figure 5) could be fit to the extended-IH model, which predicts the following relation, which is based on the reversible adiabatic compression of an ideal gas, plus the assumption that the probability of nucleation is proportional to the bubble volume:
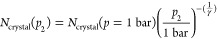
2where *N*_crystal_(*p*2) is the number of observed crystals per mL at a pressure, *p*_2_, above 1 bar, and γ = *C*_p_/*C*_v_ is the ratio of the heat capacities at constant pressure and constant volume for water vapor. It is hard to imagine a mechanism for NPLIN other than bubble formation that fits this plot. It is also stronger evidence than observing nanobubbles directly, because bubbles could be a side-product of NPLIN and not an essential element of its mechanism.

After this Perspective was submitted for publication, a paper was published by Barber and Alexander reporting the direct observation of thermocavitation events during NPLIN in aqueous CsCl using high-speed imaging with 4 μs time resolution, and that “crystals were observed to grow at the locations of the cavitation events”.^57^ This report provides compelling complementary support of the IH mechanism using a completely different experimental technique than microfluidic NPLIN. The sequential observation of vapor bubble formation and crystal growth is direct evidence that these two features are correlated, but does not prove a cause-effect relationship between bubbles and crystals. The pressure dependent studies, on the other hand, show that suppressing bubble formation suppresses crystal formation, basically showing that without bubbles, there are no crystals. Together, these two studies have solved the nearly thirty-year mystery as to the correct mechanism of NPLIN.

Looking back, we discounted the possibility of a mechanism involving the absorption of light because the bulk absorption coefficient of the solution so small that it could only heat up the solution by a few degrees. We failed to imagine that a low concentration of highly absorbing impurity particles could cause local heating by hundreds of degrees. Since the IH mechanism is purely physical (as opposed to chemical) mechanism, it is still correct to use the adjective “nonphotochemical” in the acronym NPLIN.

## Process Design and Industrial Application

We believed from the inception of microfluidic NPLIN that understanding of the mechanism(s) is a gateway to its application. Industrial crystallizations, technically challenging yet vital to a broad cross-section of societal applications, also account for significant carbon emissions. Continuous flow crystallizations have emerged in recent years for their potential to improve selectivity and properties, while mitigating the use of large quantities of solvents and energy in heating/cooling processes.^58^ The idea of controlling industrial crystallizations by understanding their mechanisms, imposing a light field and/or introducing colloidal nanoparticles, is attractive for similar reasons and especially in fields where the materials are high value, such as proteins, semiconductors, catalysts, fine chemicals and pharmaceuticals, to name a few. Continuous flow manufacturing is the most plausible approach, whereby all of the process fluids are exposed to the light, and colloidal seeds may be intentionally introduced in flow with precision. Process safety further motivates a continuous flow design, as the liquid hold-ups are minimized in industries where the use of large batch vessels account for the vast majority of chemical process accidents. That said, the potential applications of NPLIN extend beyond materials manufacturing. Lab-on-a-chip devices or micro-total-analysis systems, which perform multiple operations on chip for analyses, could benefit from scaled down and more selective purifications by NPLIN. Our goal of pinpointing the mechanism and its role in quantitative crystallizations is critical to many industrial applications.

## Future of NPLIN

While the extended-IH model is in quantitative agreement with the number of crystals we observed as a function of laser intensity, nanoparticle concentration and pressure, as well as explaining the intensity threshold, it does not explain the light polarization effects that have been reported.^4,11,12,27,29,32^ It could be that the optical electric field has a secondary effect on nucleation. More statistically significant measurements of the reported polarization effects could be carried out using continuous flow microfluidic NPLIN, with *in situ* determination of crystal size, orientation, and morphology. The work of Korede et al. applied deep learning methods to the analysis of the >1000 slugs that passed through their microfluidic device, to count slugs and to determine which ones contained crystals.^51^ Such methods could also be applied to automate the counting of crystals and determining their sizes and shapes in continuous-flow microfluidic experiments.

While pressure studies at 51.7 bar have been shown to reduce the nucleation yield by 95%, it would be interesting to study higher pressures up to the critical pressure of water at 221 bar, to see if NPLIN is completely switched off, or if there is some residual nucleation occurring even at that pressure. If there is, then one would need to characterize its behavior to determine its mechanism. The threshold intensity for NPLIN should depend on the nature of the impurity particles. Based on the IH model, the threshold should increase with increasing pressure, becoming infinite at the critical pressure.

While the IH model was developed to explain NPLIN with unfocused nanosecond laser pulses, cavitation is known to be involved in laser-induced nucleation studies using higher laser intensities (GW/cm^2^).^49,50^ Applying the IH model in this regime leads to the prediction that such studies would exhibit a pressure dependence similar to that seen in the MW/cm^2^ regime. It would be interesting to design microfluidic experiments to test this prediction.

Developing *in situ* methods to use with microfluidic NPLIN that can help to identify polymorphs, such as Raman scattering, X-ray scattering and electron microscopy,^55^ should be a priority. Computer-aided NPLIN is where the field could evolve once the preceding methods are mature. On-the-fly data analyses, in industrial and research scenarios, could go a long way toward minimizing waste and improving the desired properties and yields.
